# Inequalities in Implementation and Different Outcomes During the Growth of Laparoscopic Colorectal Cancer Surgery in England: A National Population-Based Study from 2002 to 2012

**DOI:** 10.1007/s00268-018-4615-9

**Published:** 2018-04-09

**Authors:** B. E. Byrne, C. A. Vincent, O. D. Faiz

**Affiliations:** 1 Imperial Patient Safety Translational Research Centre, Imperial College London, London, UK; 2 Bristol Centre for Surgical Research, Population Health Sciences, Bristol Medical School, University of Bristol, Canynge Hall, 39 Whatley Road, Bristol, BS8 2PS UK; 3 Department of Experimental Psychology, University of Oxford, Oxford, UK; 4 Surgical Epidemiology, Trials and Outcome Centre, St Mark’s Hospital, Harrow, UK

## Abstract

**Aim:**

Laparoscopic colorectal cancer surgery has developed from unproven technique to mainstay of treatment. This study examined the application and relative outcomes of laparoscopic and open colorectal cancer surgery over time, as laparoscopic uptake and experience have grown.

**Methods:**

Adults undergoing elective laparoscopic and open colorectal cancer surgery in the English NHS during 2002–2012 were included. Age, sex, Charlson Comorbidity Index and Index of Multiple Deprivation were compared over time. Post-operative 30-day mortality, length of stay, failure to rescue reoperation and the associated mortality rate were examined.

**Results:**

Laparoscopy rates rose from 1.1 to 50.8%. Patients undergoing laparoscopic surgery had lower comorbidity by 0.24 points (95% confidence intervals (CI) 0.20–0.27) and lower socioeconomic deprivation by 0.16 deciles (95% CI 0.12–0.20) than those having open procedures. Overall mortality fell by 48.0% from 2002–2003 to 2011–2002 and was 37.8% lower after laparoscopic surgery. Length of stay and mortality after surgical re-intervention also fell. However, re-intervention rates were higher after laparoscopic procedures by 7.8% (95% CI 0.9–15.2%).

**Conclusions:**

There was clear and persistent inequality in the application of laparoscopic colorectal cancer surgery during this study. Further work must explore and remedy inequalities to maximise patient benefit. Higher re-intervention rates after laparoscopy are unexplained and differ from randomized controlled trials. This may reflect differences in surgeons and practice between research and usual care settings and should be further investigated.

**Electronic supplementary material:**

The online version of this article (10.1007/s00268-018-4615-9) contains supplementary material, which is available to authorized users.

## Introduction

After the first description of laparoscopic colonic resection [1], concerns about oncological outcomes and port-site metastases stalled its adoption [2]. Multiple randomised controlled trials (RCTs) were subsequently conducted, reporting short-term results during 2002–2005 [3–6]. Thereafter, the National Institute for Health and Clinical Excellence for England and Wales (NICE) accepted the safety of laparoscopic colorectal surgery, recommending that this approach should be offered to patients [7]. A national training program was established in 2008 to introduce laparoscopic colorectal surgery across the country [8]. Over 50% of patients are now undergoing laparoscopic surgery for colorectal cancer in England and Wales [9].

This period provides an opportunity to examine how a new surgical technique has been introduced across a national healthcare system. During the early stages, laparoscopy may have been applied selectively. Once established, case selection should have reduced or disappeared and should only occur on clinical grounds. Previous studies have suggested that patients undergoing laparoscopic surgery tend to have lower comorbidity [10–12] or different socioeconomic characteristics [13]. However, no previous research has investigated the application and outcomes of laparoscopic colorectal cancer surgery, relative to the open approach, over time during its transition from an unproven innovation to a mainstay in the current treatment of colorectal cancer.

## Materials and methods

### Data sources

The Hospital Episode Statistics (HES) database contains diagnosis [14] and procedure codes [15] with associated dates for in-patient activity from all English National Health Service (NHS) hospitals. Statutory records of death can be linked to determine survival after surgery. From this database, patients aged 18 or more undergoing elective colorectal resection for colorectal cancer between 1 April 2002 and 31 March 2012 were identified using a combination of diagnostic codes for colorectal cancer (C18-20) and procedure codes for colorectal resections (supplementary material Table 1).

### Data processing

Duplicates were removed, and the first resection was selected where a patient underwent more than one eligible procedure. Year was aligned to the financial calendar, and laparoscopic access was coded as indicated in supplementary Table 2. Charlson Comorbidity Index (CCI) was derived from ICD-10 diagnosis codes [16, 17]. Socioeconomic deprivation was determined using the Index of Multiple Deprivation (IMD) decile. The outcomes examined were 30-day mortality, length of in-hospital stay (LOS), ‘failure to rescue-surgical’ (FTR-S) interventions within 28 days of the index procedure, and FTR-S associated in-hospital mortality. A FTR-S procedure indicates a major post-operative complication necessitating surgical correction [18]. A list of relevant OPCS codes indicating FTR-S re-interventions is provided in supplementary material Table 3.

### Data analysis

The proportion of laparoscopic operations, and the proportion completed laparoscopically or converted to open, was determined for each year. The application of laparoscopic surgery was investigated according to patient age, sex, CCI and deprivation level. These were separately modelled as dependent variables, with year and surgical approach as independent variables in multiple regression to explore changes over time and differences between laparoscopic and open. Differential change over time was assessed using an interaction between year and approach. Unadjusted annual mortality, median LOS, FTR-S re-intervention rates and FTR-S associated in-hospital mortality were compared by surgical access similarly. Logistic regression was used to examine mortality, FTR-S re-intervention and FTR-S mortality, whereas linear regression of the natural logarithm was examined for length of stay. A sensitivity analysis was conducted with risk-adjusted outcomes, using the following variables: patient age; sex; comorbidity; and anatomical site of the surgical resection.

### Ethics

The authors hold ethical approval for healthcare quality and outcomes research from the London-Queen Square Research Ethics Committee (ref: 13/LO/1235) and from the National Information Governance Board for Health and Social Care under Sect. 251 of the NHS Act 2006.

## Results

### Adoption

**Fig. 1:**
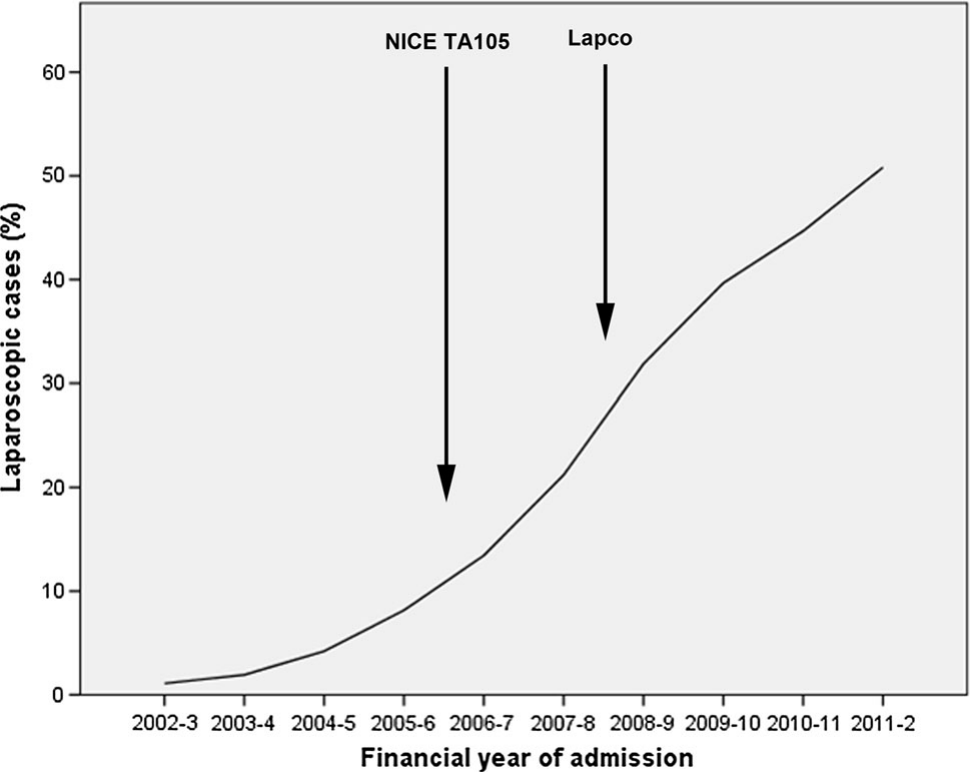
Proportion of laparoscopic cases by year of study. NICE TA105—National Institute for Health and Care Excellence Technology Appraisal 105 [7]; Lapco—National Training Program in Laparoscopic Colorectal surgery [8]

The proportion of laparoscopic cases rose from 124 (1.1%) of 12 216 in 2002–2003, to 7 391 (50.8%) of 14 543 in 2011–2002 (Table 1, Fig. 1). The proportion of cases undergoing conversion from laparoscopic to open was 4 375 (14.1%) of 31 073, and did not change over time.

**Table 1:** Number of laparoscopic and open cases by year of study, with subgroup of laparoscopic completed and converted procedures in italics

	2002–2003		2003–2004		2004–2005		2005–2006		2006–2007		2007–2008		2008–2009		2009–2010		2010–2011		2011–2012		Total	
	*n*	%	*n*	%	*n*	%	*n*	%	*n*	%	*n*	%	*n*	%	*n*	%	*n*	%	*n*	%	*n*	%
Open	12,082	98.9	11,716	98.1	11,603	95.8	12,057	91.8	11,337	86.6	10,779	78.8	9853	68.1	8703	60.3	8358	55.3	7152	49.2	103,640	76.9
Laparoscopic	134	1.1	230	1.9	510	4.2	1070	8.2	1759	13.4	2900	21.2	4609	31.9	5721	39.7	6749	44.7	7391	50.8	31,073	23.1
Completed	109	81.3	204	88.7	453	88.8	921	86.1	1514	86.1	2499	86.2	3966	86.0	4951	86.5	5785	85.7	6296	85.2	26,698	85.9
Converted	25	18.7	26	11.3	57	11.2	149	13.9	245	13.9	401	13.8	643	14.0	770	13.5	964	14.3	1095	14.8	4375	14.1
Total	12,216		11,946		12,113		13,127		13,096		13,679		14,462		14,424		15,107		14,543		134,713

### Application

**Fig. 2:**
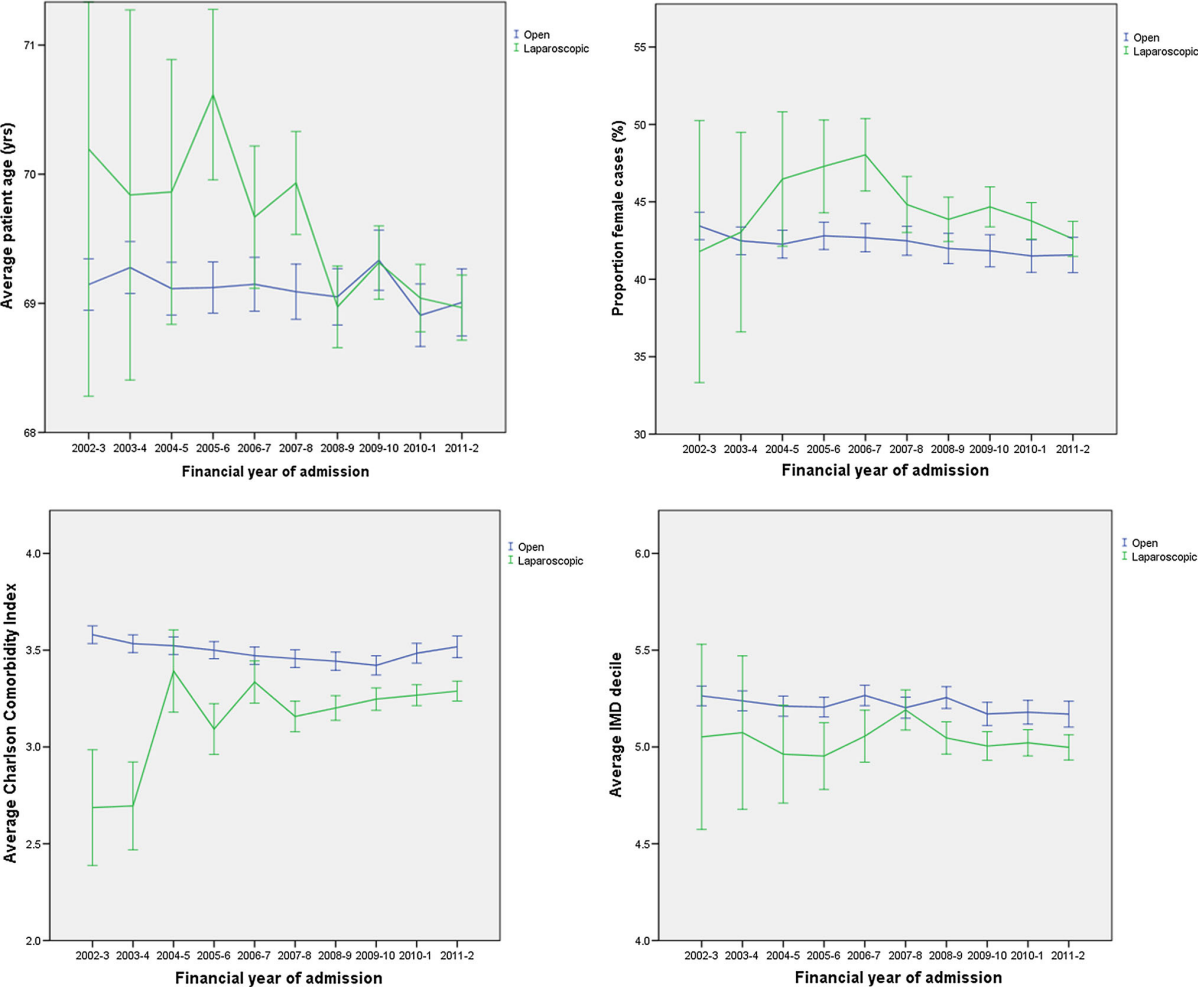
Application of laparoscopic and open surgery according to patient characteristics by year of procedure with 95% confidence intervals. *IMD* Index of Multiple Deprivation

The average patient age was 69.2 years, falling slightly over time (Fig. 2). In 2011–2002, the average patient was 0.29 years (95% confidence intervals (CI) = 0.02–0.57, *p *= 0.04) younger than in 2002–2003. Patients treated laparoscopically were, on average, 0.25 years (CI 0.09–0.40, *p* = 0.002) older than those receiving open surgery. This difference in age between groups changed over time (interaction term F change = 3.22, *df* = 9, *p* = 0.001). Over the last 4 years of the study, there was no difference in age according to surgical approach (Table 2).

**Table 2:** Characteristics of patients in laparoscopic and open groups by year of study

	2002–2003		2003–2004		2004–2005		2005–2006		2006–2007		2007–2008		2008–2009		2009–2010		2010–2011		2011–2012		Total	
	n	%	n	%	n	%	n	%	n	%	n	%	n	%	n	%	n	%	n	%	n	%
Average age (yrs)																						
Lap	70.2		69.8		69.9		70.6		69.7		69.9		69.0		69.3		69.0		69.0		69.3	
Open	69.1		69.3		69.1		69.1		69.1		69.1		69.1		69.3		68.9		69.0		69.1	
Lap																						
Male	78	58.2	131	57.0	273	53.5	564	52.7	914	52.0	1600	55.2	2587	56.1	3165	55.3	3795	56.2	4242	57.4	17,349	55.8
Female	56	41.8	99	43.0	237	46.5	506	47.3	845	48.0	1300	44.8	2022	43.9	2556	44.7	2954	43.8	3149	42.6	13,724	44.2
Open																						
Male	6833	56.6	6739	57.5	6699	57.7	6896	57.2	6497	57.3	6200	57.5	5716	58.0	5062	58.2	4889	58.5	4179	58.4	59,710	57.6
Female	5249	43.4	4977	42.5	4904	42.3	5161	42.8	4840	42.7	4579	42.5	4137	42.0	3641	41.8	3469	41.5	2973	41.6	43,930	42.4
Average CCI																						
Lap	2.69		2.70		3.39		3.09		3.34		3.16		3.20		3.25		3.27		3.29		3.24	
Open	3.58		3.53		3.52		3.50		3.47		3.46		3.44		3.42		3.48		3.52		3.50	
Average IMD decile																						
Lap	5.05		5.07		4.96		4.95		5.06		5.19		5.05		5.00		5.02		5.00		5.03	
Open	5.26		5.24		5.21		5.21		5.27		5.20		5.26		5.17		5.18		5.17		5.22	

*Lap* laparoscopic, *CCI* Charlson Comorbidity Index, *IMD* Index of Multiple Deprivation

Across the study period, 13 724 (44.2%) of 31 073 laparoscopic resections were performed in women, whereas 43 930 (42.4%) of 103 640 open procedures were performed in female patients (Table 2, Fig. 2). Over later study years, the proportion of men having a colorectal resection increased. In 2011–2012, surgical patients were 9.0% (CI 5.2–14.3%, *p *< 0.001) more likely to be male than in 2002–2003. Relative to open surgery, patients undergoing laparoscopic surgery were 10.5% (CI 7.4–13.6%, *p* < 0.001) less likely to be male. This relationship did not change over time (interaction term Chi-square = 12.15, *df* = 9, *p* = 0.21).

Average CCI fell slightly over later study years, from 3.57 in 2002–2003 to 3.52 in 2011–2012, though this did not reach significance in the final year of the study (*p* = 0.10). Patients undergoing laparoscopic surgery had lower CCI than those undergoing open surgery by 0.24 points (CI 0.20–0.27, *p* < 0.001; see Fig. 2). This relationship changed over time (interaction term F change = 4.05, *df* = 9, *p* < 0.001), although CCI was still lower among patients receiving laparoscopic surgery in 2011–2012.

The level of deprivation among patients receiving a resection for colorectal cancer fell from an average IMD decile of 5.26 to 5.17 between 2002–2003 and 2011–2012 (*p *= 0.008). Patients undergoing a laparoscopic operation had lower levels of deprivation by 0.16 deciles (CI 0.12–0.20, *p* < 0.001; see Fig. 2). This difference in IMD by operative approach did not change significantly during the study period (F change = 1.01, *df* = 9, *p* = 0.43).

### Outcomes

**Fig. 3:**
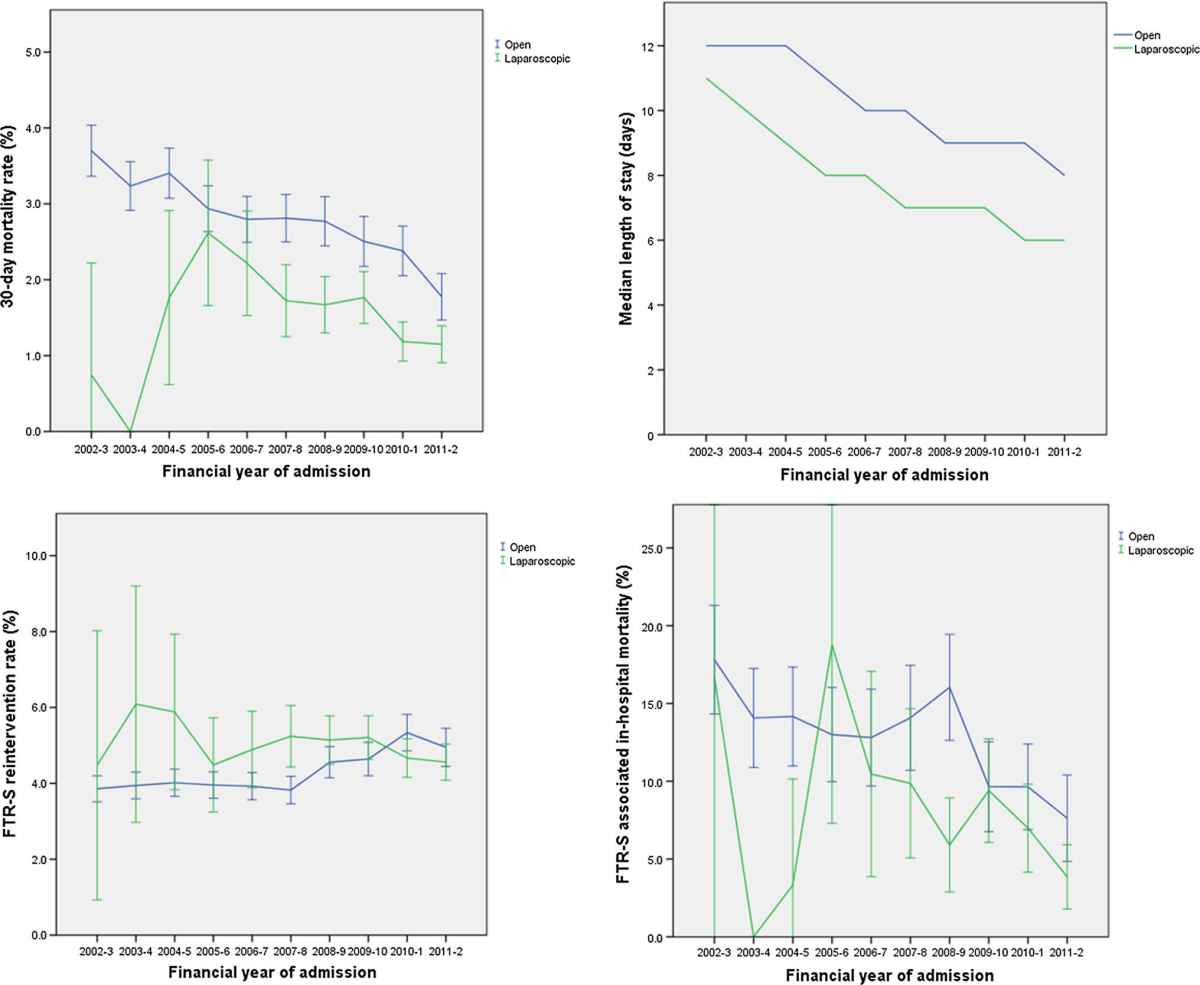
Outcomes of laparoscopic and open surgery by year of procedure with 95% confidence intervals. *FTR-S* failure to rescue-surgical

The 30-day mortality rate after laparoscopic and open surgery was 470 (1.5%) of 31 073 and 3 012 (2.9%) of 103,640, respectively (Table 3). The mortality rate after surgery fell over later study years, regardless of operative approach (Fig. 3). Due to the small sample size and infrequent event rate, statistical significance testing excluded the first 2 years of the study, after which annual numbers of laparoscopic procedures rose above 500 cases. From 2004–2005 to 2011–2012, mortality fell by 48.0% (95% confidence intervals (CI) 38.3–56.3%, *p* < 0.001). Averaged across the entire study period, the mortality rate after laparoscopic surgery was 37.8% (CI 29.7–43.2%, *p* < 0.001) lower than after open surgery. Addition of the interaction term demonstrated no change in the relationship between mortality and approach over time (Chi-square = 9.30, *df* = 7, *p* = 0.23).

**Table 3:** Outcomes for laparoscopic and open patient groups by year of study. For simplicity, totals for each group omitted from table

	2002–2003		2003–2004		2004–2005		2005–2006		2006–2007		2007–2008		2008–2009		2009–2010		2010–2011		2011–2012		Total	
	*n*	%	*n*	%	*n*	%	*n*	%	*n*	%	*n*	%	*n*	%	*n*	%	*n*	%	*n*	%	*n*	%
30-day mortality rate																						
Lap																						
Dead	1	0.7	0	0.0	9	1.8	28	2.6	39	2.2	50	1.7	77	1.7	101	1.8	80	1.2	85	1.2	470	1.5
Alive	133	99.3	230	100.0	501	98.2	1042	97.4	1720	97.8	2850	98.3	4532	98.3	5620	98.2	6669	98.8	7306	98.8	30,603	98.5
Open																						
Dead	447	3.7	379	3.2	395	3.4	354	2.9	317	2.8	303	2.8	273	2.8	218	2.5	199	2.4	127	1.8	3012	2.9
Alive	11,635	96.3	11,337	96.8	11,208	96.6	11,703	97.1	11,020	97.2	10,476	97.2	9580	97.2	8485	97.5	8159	97.6	7025	98.2	100,628	97.1
Median LOS (days)																						
Lap	11		10		9		8		8		7		7		7		6		6		7	
Open	12		12		12		11		10		10		9		9		9		8		11	
FTR-S intervention																						
Lap																						
Re-intervention	6	4.5	14	6.1	30	5.9	48	4.5	86	4.9	152	5.2	237	5.1	298	5.2	315	4.7	337	4.6	1523	4.9
None	128	95.5	216	93.9	480	94.1	1022	95.5	1673	95.1	2748	94.8	4372	94.9	5423	94.8	6434	95.3	7054	95.4	29,550	95.1
Open																						
Re-intervention	466	3.9	462	3.9	466	4.0	477	4.0	445	3.9	412	3.8	449	4.6	404	4.6	446	5.3	354	4.9	4381	4.2
None	11,616	96.1	11,254	96.1	11,137	96.0	11,580	96.0	10,892	96.1	10,367	96.2	9404	95.4	8299	95.4	7912	94.7	6798	95.1	99,259	95.8
FTR-S mortality																						
Lap																						
Dead	1	16.7	0	0.0	1	3.3	9	18.8	9	10.5	15	9.9	14	5.9	28	9.4	22	7.0	13	3.9	112	7.4
Alive	5	83.3	14	100.0	29	96.7	39	81.3	77	89.5	137	90.1	223	94.1	270	90.6	293	93.0	324	96.1	1411	92.6
Open																						
Dead	83	17.8	65	14.1	66	14.2	62	13.0	57	12.8	58	14.1	72	16.0	39	9.7	43	9.6	27	7.6	572	13.1
Alive	383	82.2	397	85.9	400	85.8	415	87.0	388	87.2	354	85.9	377	84.0	365	90.3	403	90.4	327	92.4	3809	86.9

*Lap* laparoscopic, *LOS* length of stay, *FTR-S* failure to rescue-surgical

Median post-operative length of stay fell after both laparoscopic and open surgery, from 11 and 12 days in 2002–2003, to 6 and 8 days in 2011–2012, respectively. Overall LOS fell by 32.9% (CI 32.0–33.8%, *p *< 0.001) from 2002–2003 to 2011–2012. LOS after laparoscopic surgery was 28.2% (CI 27.6–33.8%, *p* < 0.001) shorter than after open surgery. Assessment of the interaction revealed that the relationship between LOS and surgical access approach changed during the study (F change = 4.78, *df *=9, *p* < 0.001).

The rate of FTR-S intervention after laparoscopic and open surgery averaged 4.9% and 4.2%, respectively. The rate of re-intervention rose by 19.5% (CI 5.5–35.3%, *p* = 0.005) from 2002–2003 to 2011–2012. Overall, laparoscopic surgery was associated with a 7.8% higher rate of re-intervention (CI 0.9–15.2%, *p* = 0.03), although this difference changed over time (interaction term Chi-square = 26.0, *df* = 9, *p* = 0.002). FTR-S intervention rates after laparoscopic surgery appeared relatively static over time and were overall higher than those after open surgery except during the last 2 years of the study. Re-intervention rates after open surgery rose during the last 4 years of the study, eventually exceeding the rate of re-intervention after laparoscopic procedures (Fig. 3).

The in-hospital mortality rate after FTR-S intervention was 7.4% when the primary procedure was performed laparoscopically, compared with 13.1% after open surgery (Table 3). Statistical analysis was restricted to data from 2007–2008 onwards, when annual numbers of FTR-S intervention after laparoscopic surgery rose above 100. Compared to 2007–2008, the risk of in-hospital mortality after FTR-S intervention fell by 54.3% (CI 31.5–69.6%, *p* < 0.001) in 2011–2012, irrespective of the primary surgical approach. When the initial procedure was performed laparoscopically, FTR-S mortality was 39.4% lower (CI 22.0–53.0%, *p *< 0.001) than after an open index operation. This difference between patient groups, according to the initial operative approach, did not change over time (interaction term Chi-square = 8.51, *df* = 4, *p* = 0.07).

Sensitivity analyses of risk-adjusted outcomes did not result in significant changes to any of the above results.

## Discussion

This study presents evidence of clear and persistent inequality in the application laparoscopic techniques for colorectal cancer surgery in the English NHS between 2002 and 2012. Despite uptake reaching 50.8% in 2011–12, laparoscopic resection continued to be associated with lower comorbidity and socioeconomic deprivation. However, re-intervention rates were higher among patients undergoing laparoscopic surgery, despite the lower risk profile of this patient population.

This study has important, well-described strengths and limitations associated with the use of national administrative datasets. These tend to capture a more complete picture of national activity than voluntary registers [19, 20], have been shown to permit accurate modelling of patient outcomes [21] and allow identification of clinically important events [22]. Limitations include the lack of data on cancer stage, which may have had implications for patient selection, although this should have had limited impact on 30-day outcomes of elective resection. This study included both colonic and rectal cancer surgery, and rectal surgery is considered more technically challenging. This could have affected patient selection. However, sensitivity analysis of separate colonic and rectal surgical groups did not alter the key findings of this study regarding comorbidity, socioeconomic deprivation, mortality and length of stay. Another limitation of this study was the focus on unadjusted outcomes. However, this was a deliberate choice. This study has highlighted differences in the populations of patients undergoing laparoscopic and open surgery across measured characteristics. It is therefore likely that these two patient populations were also different across unmeasured characteristics, which cannot be controlled for using statistical techniques. Appropriately interpreted, unadjusted outcomes may yield important insights and reveal granular changes in trends that may be hidden as average effects in multiple regression analysis.

Differential application of laparoscopic surgery persisted throughout the study period, despite the accumulation of experience and attainment of high levels of adoption in later study years. Previous, non-longitudinal research in Canada [10] and the USA [11, 12] also found an association between laparoscopic surgery and lower comorbidity. Differences in comorbidity and socioeconomic status between laparoscopic and open patient groups may have arisen at the surgeon level, through case selection, or at the unit level, through geographic variation. During early adoption, novice surgeons may have selected ‘easier’, less comorbid patients for whom a prolonged operation and anaesthetic should not cause untoward problems. With progression along the learning curve, it may reasonably be expected that surgeons would apply the technique to all suitable patients. The narrowing of the differences in comorbidity in the early years of this study may support this contention. However, lower comorbidity and deprivation in the laparoscopic group stubbornly persisted over the latter half of the study. Differences in application may have occurred at the unit level, with surgeons trained in laparoscopic techniques perhaps practicing in less socioeconomically deprived areas, where patients are also likely to be less comorbid.

Further research to specifically explore this finding should be conducted, across other types of surgery and in other healthcare systems. The level at which selection is occurring needs to be determined, as this may have important clinical, ethical and policy implications. If laparoscopic surgery is associated with improved outcomes, surgeons have a moral duty to ensure that all suitable patients benefit from this approach. There may also be ramifications for policy makers, to ensure the benefits of laparoscopic surgery are delivered widely across the healthcare system. Analysis of more recent data is also needed to establish whether differences in application have persisted beyond the end of the study period, as laparoscopic surgery has become even more embedded into routine practice.

The lower mortality rate after laparoscopic compared with open surgery is consistent with other large observational studies of colorectal surgery [23, 24]. However, data from RCTs have shown no difference in mortality rates between the two operative approaches [3–6], with a number of systematic reviews and meta-analyses reaching the same conclusion [25–27]. The present study has already discussed clear evidence of patient selection for laparoscopic surgery, and the lower levels of comorbidity and deprivation among laparoscopically treated patients may be key explanatory factors for the lower mortality rates observed. RCTs are designed to tackle biases due to patient selection, and findings from such study designs take priority over observational research in determining whether any survival benefit may be attributed to the laparoscopic approach.

Higher re-intervention rates after laparoscopic surgery in the present study also contrast with data from RCTs and meta-analyses, which have reported comparable or lower complication rates associated with the laparoscopic technique [3–5, 26]. The authors are unaware of any other studies presenting a similar finding. While further work is required specifically to explore this finding, we propose some possible explanations. Surgeons who self-select to participate within RCTs may have greater laparoscopic experience or above average laparoscopic skill, and be enthusiasts for the technique. Conversely, the wider population of surgeons represented in this study may have had less experience, or simply represent the average level of surgical skill, with associated higher complication rates. In addition, clinical care within an RCT may be more structured and closely monitored than usual clinical care, resulting in lower complication rates. During the study period, there may also have been changes in the management of post-operative complications within the surgical community. Research has suggested that a key determinant of outcome is not necessarily the rate of complications, but the ability to successfully ‘rescue’ patients when complications occur [18, 28]. When learning laparoscopic surgery, surgeons may have had a higher index of suspicion for complications and had a lower threshold to investigate and treat during the post-operative period. Over time, experience with successful rescue may have consolidated an aggressive approach to complication management as a standard of care after both laparoscopic and open surgery. This study provides tentative support for this argument, as the rate of re-intervention after open surgery rose during later years of the study.

It is interesting to note that the conversion rate from laparoscopic to open did not change during the study period. This may arise from the population-level nature of this study. While individual surgeons will have had demonstrable learning curves, gradual introduction of laparoscopy will have resulted in staggering of these learning curves over several years, smoothing out the surgeon-level effect on outcomes. The stable conversion rate may also suggest that the surgical profession has been effective in managing the introduction of laparoscopic techniques without compromising care during the early learning curve. For example, the national training program in laparoscopic colorectal surgery instituted a structured process of mentoring to allow supervised development of laparoscopic skills by existing consultants keen to learn this technique. Data on participation in the program are not available within HES to explore the role of this program in more detail.

Overall, this study has documented substantial improvements in the outcomes of all patients, regardless of the operative approach, with a 48.0% fall in 30-day mortality from 2004–2005 to 2011–2012, exceeding the survival benefit associated with laparoscopic surgery. Length of stay has also fallen significantly for all patients. These improvements may owe to a wide range of improvements in all relevant aspects of modern medical and surgical care. In particular, the reduction in post-operative length of stay may have been driven by widespread adoption of Enhanced Recovery After Surgery (ERAS) protocols [29]. Beyond this, there may have been improvements in medical optimisation of patients and more effective perioperative management, such as higher quality intensive care [30, 31].

The present findings should stimulate further research into patterns of uptake in other fields of minimal access surgery, and in other healthcare systems. Specific considerations relevant to colorectal cancer surgery, including screening and the national laparoscopic training program, may have influenced the findings presented, potentially limiting generalisation to other settings.

This study has shown that significant inequality in the utilisation of laparoscopy for colorectal cancer has persisted despite high levels of adoption, meaning that the benefits of the laparoscopic approach are not yet being fully realised within the NHS as a whole. Mortality and length of stay outcomes improved dramatically after both laparoscopic and open surgery during this ten-year study. However, the rate of re-intervention after laparoscopic surgery was higher than after open surgery, an unexpected finding that requires further examination. It is appropriate that future innovations and new techniques may be selectively applied in their early stages, but long-term population- or disease-based studies will be required to ensure medical advances are applied equitably to achieve the greatest benefit for patients.

## Electronic supplementary material

Below is the link to the electronic supplementary material.

Supplementary material 1 (DOCX 29 kb)

## References

[1] Jacobs M, Verdeja JC, Goldstein HS (1991). Minimally invasive colon resection (laparoscopic colectomy). Surg Laparosc Endosc.

[2] Senagore AJ (2015). Adoption of laparoscopic colorectal surgery: it was quite a journey. Clin Colon Rectal Surg.

[3] Guillou PJ, Quirke P, Thorpe H (2005). Short-term endpoints of conventional versus laparoscopic-assisted surgery in patients with colorectal cancer (MRC CLASICC trial): multicentre, randomised controlled trial. Lancet.

[4] The COlon cancer Laparoscopic or Open Resection (COLOR) Study Group (2005). Laparoscopic surgery versus open surgery for colon cancer: short-term outcomes of a randomised trial. Lancet Oncol.

[5] Lacy AM, García-Valdecasas JC, Delgado S (2002). Laparoscopy-assisted colectomy versus open colectomy for treatment of non-metastatic colon cancer: a randomised trial. Lancet.

[6] The Clinical Outcomes of Surgical Therapy Study Group (2004). A comparison of laparoscopically assisted and open colectomy for colon cancer. N Engl J Med.

[7] National Institute for Health and Care Excellence (2006) Laparoscopic surgery for colorectal cancer: technology appraisal guidance. https://www.nice.org.uk/guidance/ta105

[8] Coleman MG, Hanna GB, Kennedy R (2011). The national training programme for laparoscopic colorectal surgery in England: a new training paradigm. Color Dis.

[9] Healthcare Quality Improvement Partnership Ltd (2016) National bowel cancer audit annual report

[10] Chan BP, Gomes T, Musselman RP (2012). Trends in colon cancer surgery in Ontario: 2002–2009. Color Dis.

[11] Taylor EF, Thomas JD, Whitehouse LE (2013). Population-based study of laparoscopic colorectal cancer surgery 2006–2008. Br J Surg.

[12] Kwon S, Billingham R, Farrokhi E (2012). Adoption of laparoscopy for elective colorectal resection: a report from the surgical care and outcomes assessment program. J Am Coll Surg.

[13] Bardakcioglu O, Khan A, Aldridge C, Chen J (2013). Growth of laparoscopic colectomy in the United States: analysis of regional and socioeconomic factors over time. Ann Surg.

[14] World Health Organization (1992). International statistical classification of diseases and related health problems (tenth revision).

[15] Information Standards Board for Health and Social Care (2014) OPCS 4.7—next version. http://systems.hscic.gov.uk/data/clinicalcoding/codingstandards/opcs4/opcs-4.7. Accessed 15 June 2016

[16] Quan H, Sundararajan V, Halfon P (2005). Coding algorithms for defining comorbidities in ICD-9-CM and ICD-10 administrative data. Med Care.

[17] Charlson ME, Pompei P, Ales KL, MacKenzie CR (1987). A new method of classifying prognostic comorbidity in longitudinal studies: development and validation. J Chronic Dis.

[18] Almoudaris AM, Burns EM, Mamidanna R (2011). Value of failure to rescue as a marker of the standard of care following reoperation for complications after colorectal resection. Br J Surg.

[19] Almoudaris AM, Burns EM, Bottle A (2011). A colorectal perspective on voluntary submission of outcome data to clinical registries. Br J Surg.

[20] Aylin P, Lees T, Baker S (2007). Descriptive study comparing routine hospital administrative data with the Vascular Society of Great Britain and Ireland’s National Vascular Database. Eur J Vasc Endovasc Surg.

[21] Aylin P, Bottle A, Majeed A (2007). Use of administrative data or clinical databases as predictors of risk of death in hospital: comparison of models. Br Med J.

[22] Raleigh VS, Cooper J, Bremner SA, Scobie S (2008). Patient safety indicators for England from hospital administrative data: case-control analysis and comparison with US data. Br Med J.

[23] Faiz O, Warusavitarne J, Bottle A (2009). Laparoscopically assisted vs. open elective colonic and rectal resection: a comparison of outcomes in English National Health Service Trusts between 1996 and 2006. Dis Colon Rectum.

[24] Kang CY, Halabi WJ, Luo R (2012). Laparoscopic colorectal surgery: a better look into the latest trends. Arch Surg.

[25] Ohtani H, Tamamori Y, Arimoto Y (2011). A meta-analysis of the short- and long-term results of randomized controlled trials that compared laparoscopy-assisted and conventional open surgery for colorectal cancer. J Cancer.

[26] Wang C-L, Qu G, Xu H-W (2014). The short- and long-term outcomes of laparoscopic versus open surgery for colorectal cancer: a meta-analysis. Int J Colorectal Dis.

[27] Schwenk W, Haase O, Neudecker JJ, Müller JM (2005). Short term benefits for laparoscopic colorectal resection. Cochrane Database Syst Rev.

[28] Ghaferi AA, Birkmeyer JD, Dimick JB (2011). Hospital volume and failure to rescue with high-risk surgery. Med Care.

[29] Zhuang C-L, Ye X-Z, Zhang X-D (2013). Enhanced recovery after surgery programs versus traditional care for colorectal surgery: a meta-analysis of randomized controlled trials. Dis Colon Rectum.

[30] Kaukonen K-M, Bailey M, Suzuki S (2014). Mortality related to severe sepsis and septic shock among critically ill patients in Australia and New Zealand, 2000–2012. J Am Med Assoc.

[31] Zimmerman JE, Kramer AA, Knaus WA (2013). Changes in hospital mortality for United States intensive care unit admissions from 1988 to 2012. Crit Care.

