# Supplementary material for: Inequalities in Implementation and Different Outcomes During the Growth of Laparoscopic Colorectal Cancer Surgery in England: A National Population-Based Study from 2002 to 2012
Source: World J Surg. 2018 Apr 9;42(10):3422–31. doi: 10.1007/s00268-018-4615-9 (PMC6132863; doi:10.1007/s00268-018-4615-9)
Supplement: Supplementary file 1 — Supplementary material 1 (DOCX 29 kb) [file 268_2018_4615_MOESM1_ESM.docx]

**Supplemental material**

| Group | Description | Code |
| --- | --- | --- |
| Right colon | Extended excision of right hemicolon | H06 |
|  | Other excision of right hemicolon | H07 |
|  | Excision of transverse colon | H08 |
| Left colon | Excision of left hemicolon | H09 |
|  | Excision of sigmoid colon | H10 |
| Rectum | Excision of rectum | H33 |
| Total / subtotal | Total excision of colon and rectum | H04 |
|  | Total excision of colon | H05 |
|  | Subtotal excision of colon | H29 |
| Other | Other excision of colon | H11 |

Supplementary table 1. OPCS-4 codes for data extraction from HES.

|  | Code |
| --- | --- |
| Laparoscopic access | Y75.1-4 |
|  | Y75.8-9 |
|  | Y76.3 |
|  | Y76.5 |
|  | Y76.8-9 |
|  | Y50.8 |
|  | Y52.8 |

Supplementary table 2. OPCS-4 codes indicating laparoscopic access.

| Procedure group | OPCS codes |
| --- | --- |
| Small bowel | G49, G51, G53.2, G58, G61, G63.4, G69, G71, G72, G73, G75, G78.4 |
| Colorectal | H04, H05, H06, H07, H08, H09, H10, H11, H13, H17, H29, H33 |
| Stoma | G60, G74, G75, H14, H15 |
| Deep sepsis | T34, T45, T46.3, T46.8-9, Y22 |
| Superficial sepsis | S47.2, S47.4, S47.6, S47.8-9, T31.5 |
| Bleeding | J69, J70.1, J70.8-9, J72.2, J72.4, J72.8-9, T30.1 |
| Adhesions | T41.2-3, T41.5, T41.8-9, T42.3 |
| Other | T30.2-4, T30.8-9, T41.4, T42.4, Y29, Y31-2, Y50.2, Y70.1-2, Y75 |

Supplementary table 3. OPCS-4 codes indicating a failure to rescue – surgical procedure.
